# Prognostic value of plasma von Willebrand factor levels in major adverse cardiovascular events: a systematic review and meta-analysis

**DOI:** 10.1186/s12872-020-01375-7

**Published:** 2020-02-10

**Authors:** Mengge Fan, Xia Wang, Xun Peng, Shuo Feng, Junyu Zhao, Lin Liao, Yong Zhang, Yinglong Hou, Ju Liu

**Affiliations:** 1 Laboratory of Microvascular Medicine, Medical Research Center, Shandong Provincial Qianfoshan Hospital, The First Affiliated Hospital of Shandong First Medical University, 16766 Jingshi Road, Jinan, 250014 Shandong China; 2 Graduate School, Shandong First Medical University & Shandong Academy of Medical Sciences, Jinan, China; 3 School of Medicine, Shandong University, Jinan, China; 4 Department of Endocrinology, Shandong Provincial Qianfoshan Hospital, The First Affiliated Hospital of Shandong First Medical University, Jinan, China; 5 Department of Cardiology, Shandong Provincial Qianfoshan Hospital, The First Affiliated Hospital of Shandong First Medical University, Jinan, China

**Keywords:** Major adverse cardiovascular events, Coronary artery disease, von Willebrand factor, Meta-analysis

## Abstract

**Background:**

Prediction of major adverse cardiovascular events (MACEs) may offer great benefits for patients with coronary artery disease (CAD). Von Willebrand factor (vWF) is stored in endothelial cells and released into blood plasma upon vascular dysfunction. This meta-analysis was performed to evaluate the prognostic value of plasma vWF levels in CAD patients with MACEs.

**Methods:**

A total of 15 studies were included in this meta-analysis through the search in PubMed, Embase and CNKI. Data were collected from 960 patients who had MACEs after CAD and 3224 controls nested without the adverse events. The standard mean difference (SMD) and 95% confidence intervals (95% CI) were calculated using random-effects model.

**Results:**

The plasma vWF levels examined at 24 h and 48 h after admission were significantly higher in CAD patients with MACEs than those without. The pooled SMD among the MACEs group and the non-MACEs group was 0.55 (95% CI = 0.30–0.80, *P* < 0.0001) and 0.70 (95% CI = 0.27–1.13, *P* = 0.001), respectively. However, no significant difference was found in plasma vWF levels on admission between the two groups.

**Conclusion:**

Plasma vWF level in CAD patients examined at 24 h and 48 h after admission might be an independent prognostic factor for MACE.

## Background

Coronary artery disease (CAD) is characterized by the occlusion or stenosis of coronary artery mostly caused by atherosclerosis, and is one of the leading causes of mortality in humans [1, 2]. Patients with CAD are vulnerable in development of major cardiovascular events (MACEs) including nonfatal acute myocardial infarction, unstable angina, stroke, transient ischemic attack, peripheral arterial occlusive disorder, and death [3]. According to previous long-term follow-up studies, the incidence of MACEs development after CAD ranges from 21 to 49% [4], while the recurrence rates of MACEs are up to 75% within 3 years [5]. These events are typically caused by the formation of thrombus and insufficient blood supply. Identifying risk factors for the development of MACEs is of great value in the prognosis of CAD patients.

Von Willebrand factor (vWF) is a large multimeric glycoprotein required for the formation of hemostatic plugs and arterial thrombi [6]. vWF adheres platelets to the blood vessel wall, and acts as a plasma carrier for factor VIII to prevent its degradation in the blood circulation [7, 8]. After synthesis, vWF is stored in the Weibel-Palade bodies of endothelial cells and the α-granules of platelets [9, 10]. The stored vWF is rapidly released at moments of endothelial cell damage, thus it is considered as a promised biomarker for endothelial dysfunction [11]. In addition, studies have shown that the plasma vWF derived from coronary vascular endothelial cells is significantly elevated when coronary artery injury occurs [12], indicating a pathogenesis role of vWF in the progression of CAD. Increased level of vWF has been recognized as an independent predictor of CAD in general population [9]. However, the prognostic role of vWF in CAD patients remains controversial. Several studies demonstrate a weak association between elevated vWF level and adverse outcomes in patients with CAD [13]. The current meta-analysis was performed to evaluate the prognostic value of plasma vWF in patients with CAD, in terms of MACEs.

## Methods

### Search strategy

We searched for all publications concerning the association between vWF and CAD up to July 2018. The literatures were searched in PubMed, Embase database and CNKI. The search strategy was composed by the following search terms (vWF OR Willebrand Protein OR von Willebrand Factor OR Factor VIIIR-Ag) AND (Coronary Disease OR coronary artery disease OR CAD OR CHD OR Myocardial Infarction OR AMI OR Acute coronary syndrome OR ACS OR angina) AND (major adverse cardiac events OR mortality OR death OR prognosis).

### Study selection

Inclusion criteria were as follows: (1) cohort studies enrolling patients with CAD (myocardial Infarction, acute coronary syndrome and stable CAD); (2) data on plasma vWF was reported; (3) MACEs or mortality following CAD were recorded; (4) studies written in English or Chinese. Exclusion criteria are as follows: (1) patients without CAD; (2) there is no definitive value of plasma vWF.

### Data extraction

Two independent reviewers extracted the following data from each eligible studies: first author’s name, year of publication, mean age, sample size, gender, mean and standard deviation (mean ± SD) or mean and standard error (mean ± SE) of plasma vWF concentration, definition of MACEs, mean or median follow-up duration, treatment modality, data regarding baseline and follow-up concentrations of vWF. Moreover, if the articles provide the data of median and interquartile range (IQR) format or mean and *p* value, we calculated the SMD according to the formulations recommended by Cochrane Handbooks. Any discrepancy in data extraction was resolved through discussion with a third reviewer.

### Quality assessment

Two independent authors assessed the methodological quality according to the Newcastle-Ottawa Quality Assessment scale (NOS) for cohort study. Total NOS score ranged from 0 to 9 stars. Those scored ≥7 stars were considered as high quality and those scored ≤5 as low quality.

### Statistical analysis

Meta-analyses were conducted on Review Manager software (RevMan5.3, Cochrane Collaboration, Oxford, UK, http://community.cochrane.org) and STATA software (Stata Corp, College Station, Texas, USA). The SMD and 95% confidence intervals were calculated using a generic inverse variance approach. The overall effects were determined by Z-test and *P*-value < 0.05 were considered as statistically significant.

The heterogeneity across studies was tested by CochranQ statistics and I^2^ statistics. A random-effects model was used due to significant heterogeneity. Subgroup analyses were conducted to identify the source of potential heterogeneity based on the duration of follow-up, PCI, and severity of CAD. Meta-regression was also conducted to explore the potential heterogeneity. *P* < 0.05 was considered statistically significant. Sensitivity analysis was performed by removing studies one by one to estimate the stability of meta-analysis.

## Results

### Search results and characteristics of included studies

**Fig. 1:**
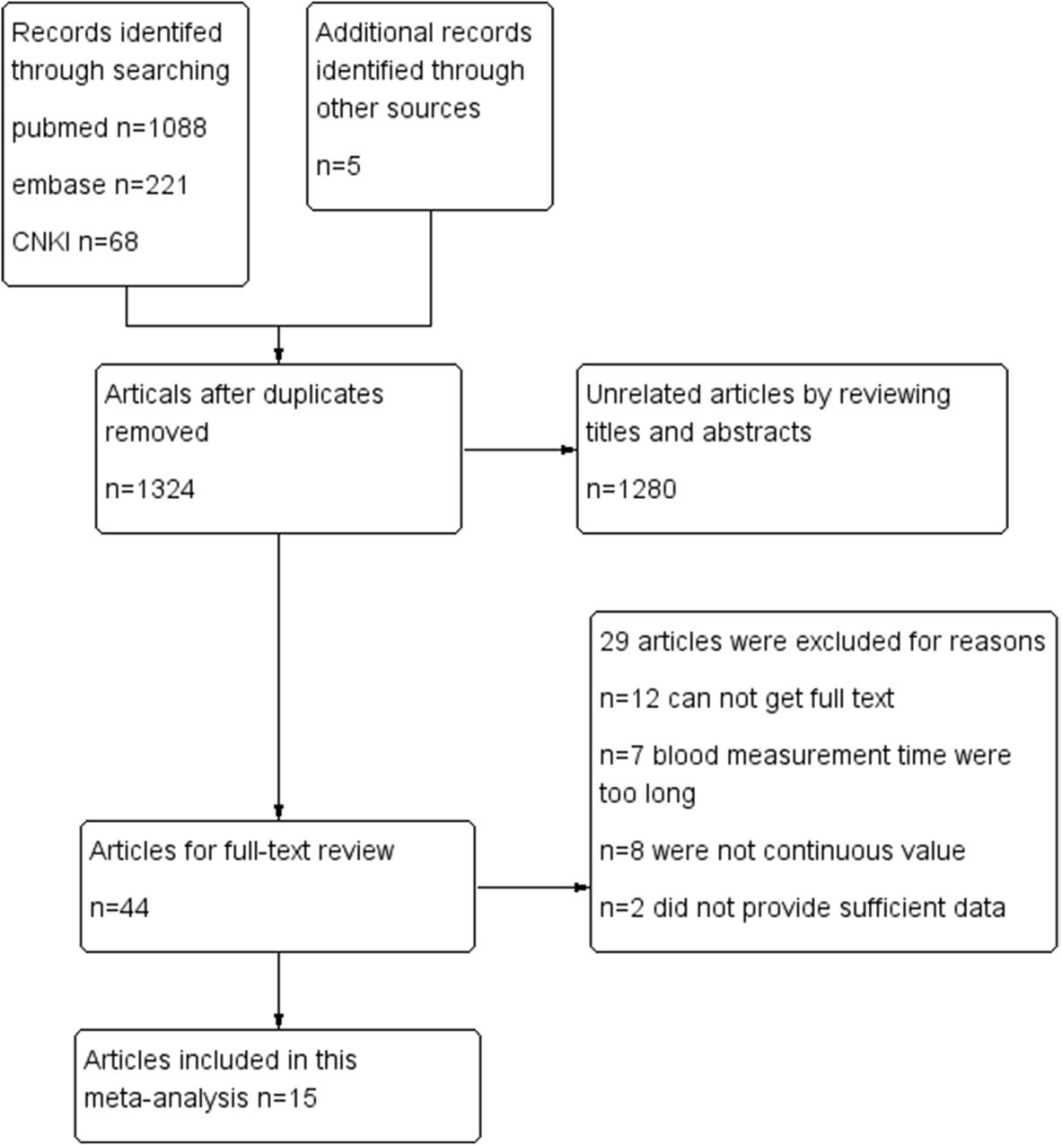
The flow chart of the literature search and selection process

A total of 1382 publications were identified by the search strategy, and 1324 publications remained in this study after removal of duplicates. After carefully reviewing the titles and abstracts, 44 candidate articles were screened out for further full-text reading, and 1280 unrelated articles were excluded. In addition, 27 full-text reviewed articles were excluded due to disqualification of inclusion criteria, 15 studies were included in this meta-analysis. Search progress was shown in Fig. 1. Of these 15 studies, 4 studies were conducted in China, 3 in Austria, 3 in UK, 2 in Germany, 1 in USA, 1 in Norway and 1 in France. A total of 4184 patients with CAD were identified and analyzed. Individual study sample sizes varied from 58 to 1045, and the duration of follow-up ranged from 30 days to 13 years. The included studies provided the plasma level of vWF at different time points after CAD (on admission, 24 h, 48 h). The main characteristics of the studies were shown in Table 1.

**Table 1:** Characteristics of the studies included in the meta-analysis

Year	Author	Blood Sampling Schedule	Patients	Age, y C/CTL	Sample size C/CTL	Treatment modality	Definition of MACEs	Measuring Methods	Rates of HF C/CTL	Follow up
2016	HAMID	24 h	STEMI	57/53	17/61	PCI, Thrombolytic	All-cause mortality, recurrent nonfatal MI, or HF and the secondary endpoint of early adverse LV remodeling	ELISA	NR	30 days
2015	Liu	Admission	STEMI	58/60	30/102	PCI	Recurrent MI, heart failure readmission, unplanned repeat revascularization, malignant dysrhythmia, stroke, or pulmonary embolism	ELISA	NR	1 year
2013	Leu	Admission	CAD	68/67	33/42	Antiplatelet	CV death, nonfatal AMI, unstable angina, stroke, transient ischemic attack, or peripheral arterial occlusive disorder	ELISA	NR	40 months
2013	Hyseni	Admission	ACS	67.5/ 76.8	293/46	PCI, Antithrombotic	All-cause mortality	ELISA	NR	4 years
2008	Yu	Admission, 12 h, 48 h	ACS	68/64	22/48	Anticoagulation	Death, MI or recurrent MI, and recurrent angina	ELISA	NR	30 days
2008	BOOS	24 h	ACS	69/60.6	42/169	Thrombolysis, PCI	CV death, non-fatal MI, readmission with acute HF and stroke, and CV death separately.	ELISA	28.6 / 7.6	338 days
2006	Fuchs	24 h	ACS	60/57	58/150	PCI, Thrombolysis	Recurrent non-fatal MI (STEMI and NSTE-MI)	Turbidometry	NR	28 months
2006	An	24 h	ACS	NR	21/59	NR	Non-fatal reinfarction, non-fatal heart failure, recurrent angina attacks, drug intensification or emergency revascularization, and cardiac death	ELISA	NR	30 days
2005	Lee	Admission, 48 h	ACS	67/70	24/34	Antiplatelet, Anticoagulation	Death, MI, and refractory angina requiring revascularisation	ELISA	8 /6	30 days
2005	Warlo	24 h	CAD	NR	73/927	Antiplatelet	Unstable angina pectoris, MI, non haemorrhagic stroke and death	NR	NR	2 years
2003	Niessner	Admission	CAD	56/52	103/38	NR	All-cause mortality and MI, revascularization procedures including PTCA with/without coronary stenting and ACBG.	ELISA	NR	13 years
2002	Eikelboom	Admission	ACS	NR	78/407	Anticoagulant, Antiplatelet	CV death, MI, stroke or refractory ischaemia	NR	NR	30 days
2000	Redondo	Admission	CAD	59/57	37/157	NR	Fatal MI, non-fatal MI, percutaneous transluminal coronary angioplasty or CABG.	ELISA	NR	2 years
1999	Moss	Admission	MI	59/47	81/964	NR	Death due to coronary heart disease or recurrent nonfatal MI	ELISA	NR	26 months
1998	Montalescot	Admission, 48 h	CAD	70/70	48/20	Antiplatelet	Death, MI, recurrent angina, or revascularization	ELISA	NR	30 days

*Abbreviations*: *ACBG* aorto coronary bypass graft, *ACS* acute coronary syndromes, *AMI* acute myocardial infarction, *CABG* coronary artery bypass grafting, *C/CTL* case/control group, *CV* cardiovascular, *ELISA* enzyme-linked immunosorbent assay, *HF* heart failure, *NR* unreported, *LV* left ventricular, *MI* myocardial infarction, *non-STEMI* non-ST-elevated myocardial infarction, *PCI* percutaneous coronary intervention, *PTCA* percutaneous transluminal coronary angioplasty, *STMI* ST-elevated myocardial infarction

### Plasma vWF and MACEs

**Fig. 2:**
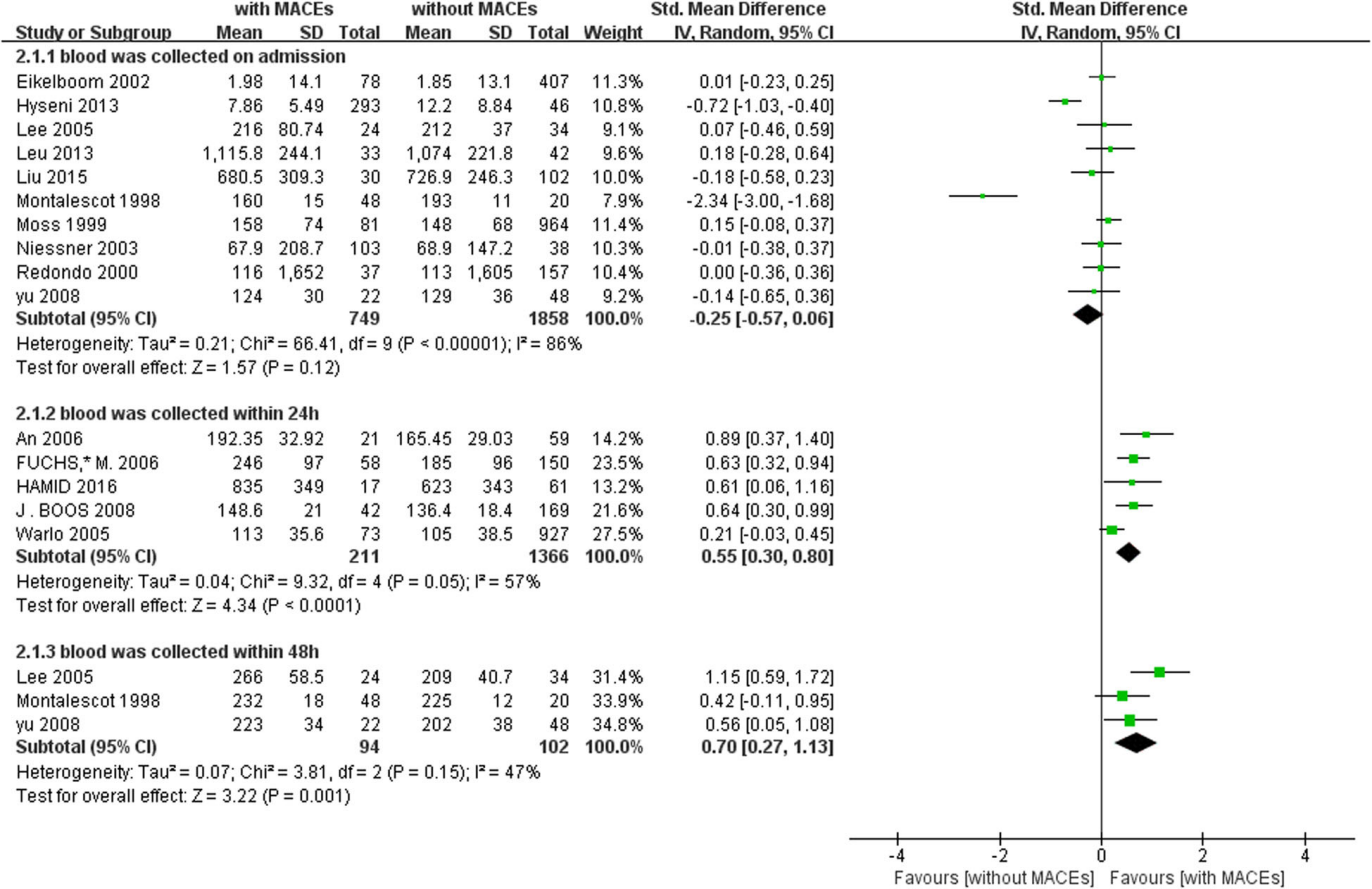
Forest plot of meta-analysis of plasma vWF after MACEs. Each block represents a study and the area of each block is proportional to the weight of that study. The horizontal line represents each study’s 95% confidence interval (CI) for the effect. The centre of the diamond is the pooled effect across studies, and the width of the diamond denotes its 95% CI. CAD, coronary artery disease; MACEs: major adverse cardiac events; IV, invers variance; SD, standard deviation

The meta-analyses were conducted according to the time points of vWF examination (on admission, 24 h and 48 h after admission). Consistent with previous reports, we found that the plasma level of vWF is elevated in CAD patients. The result of meta-analyses further to reveal that plasma level of vWF is significantly higher in CAD patients with MACEs than those without MACEs (Fig. 2). The pooled SMD for vWF examined at 24 h and 48 h after admission was 0.55 (95% CI = 0.30–0.80, *P* < 0.0001) and 0.70 (95% CI = 0.27–1.13, *P* = 0.001), respectively. However, there was no significant difference in plasma level of vWF examined on admission between the two groups. The pooled SMD was − 0.25 (95% CI = − 0.75-0.06, *P* = 0.12). In addition, heterogeneity across studies was present.

### Quality evaluation

Four studies [3, 4, 14, 15] with 8 NOS scores and eight studies [10, 16–22] with 7 NOS scores were considered as good quality. Other studies [13, 23, 24] achieved 6 scores indicating moderate quality. The results of the quality assessment of the included studies were shown in Table 2.

**Table 2:** Quality assessment of the included studies based on the Newcastle–Ottawa Scale

Author	Study design	Selection	Comparability	Outcome	Total scores
HAMID	Cohort study	3	2	2	7
Liu	Cohort study	3	2	3	8
Leu	Cohort study	3	2	3	8
Hyseni	Cohort study	3	1	2	6
yu	Cohort study	3	2	2	7
BOOS	Cohort study	3	2	3	8
Fuchs	Cohort study	2	2	2	6
An	Cohort study	2	2	2	6
Lee	Cohort study	3	2	3	8
Warlo	Cohort study	2	2	3	7
Niessner	Cohort study	3	2	2	7
Eikelboom	Cohort study	3	2	2	7
Redondo	Cohort study	3	2	2	7
Montalescot	Cohort study	2	2	3	7
Moss	Cohort study	2	2	3	7

### Heterogeneity

Subgroup analyses were conducted to evaluate potential sources of heterogeneity. Include studies were subgrouped according to the duration of follow-up. As shown in Table 3, the pooled effects of the meta-analyses were not reversed by the duration of follow-up. However, when subgrouped by the severity of CAD, a significant difference in SMD in vWF plasma levels examined at 24 h after admission was found between the subgroups. The pooled SMD was 0.67 (95% CI = 0.47–0.86, *P* < 0.00001) for acute coronary syndrome (ACS) and myocardial infarction (MI) patients and was 0.21 (95% CI = − 0.03-0.45, *P* = 0.09) for stable CAD patients. No significant difference in SMD in vWF plasma levels examined at 24 h after admission was found between patients treated with PCI (SMD = 0.63, 95% CI = 0.42–0.84, *P* < 0.00001) and those without PCI (SMD = 0.33, 95% CI = 0.11–0.54, *P* = 0.003). To validate the results from subgroup analyses, we performed meta-regression to determine the source of heterogeneity. The associations between types of coronary disease, high range of follow up sample size, patients treated with PCI and the utilization of antiplatelet or anticoagulation were evaluated. As shown in Table 4, types of coronary disease, high range of follow up sample size, patients treated with PCI, and the utilization of antiplatelet or anticoagulation were not the source of heterogeneity in vWF plasma levels examined on admission (types of coronary disease: *P* = 0.489; high range of follow up sample size: *P* = 0.364; patients treated with PCI: *P* = 0.725; the utilization of antiplatelet: *P* = 0.527; the utilization of anticoagulation drugs: *P* = 0.509). However, both high range of follow up sample size and the utilization of antiplatelet or anticoagulation contribute to heterogeneity in vWF plasma levels examined at 24 h after admission (high range of follow up sample size: *P* = 0.033; the utilization of antiplatelet or anticoagulation: *P* = 0.007).

**Table 3:** Subgroup analyses on MACEs

Subgroup		No. of studies	No. of subjects		Meta-analysis			Heterogeneity	
			MACEs	Non MACEs	SMD	95% CI	*P*	I^2^ (%)	*P*
Follow-up duration									
On admission	< 1 year	4	172	509	−0.57	−1.44-0.3	0.2	93	< 0.00001
	≥1 year	6	577	1349	−0.10	−0.39-0.19	0.5	76	0.0007
24 h	< 1 year	2	38	120	0.76	0.38–1.13	< 0.0001	0	0.47
	≥1 year	3	173	1246	0.48	0.17–0.78	0.002	69	0.04
Type of CAD									
On admission	CAD	4	221	257	−0.20	−0.42-0.01	0.06	93	< 0.00001
	ACS、MI	6	528	1601	−0.09	−0.22-0.04	0.16	76	0.04
24 h	CAD	1	73	927	0.21	−0.03-0.45	0.09		
	ACS、MI	4	138	439	0.67	0.47–0.86	< 0.00001	0	0.86
PCI									
On admission	Yes	2	323	148	−0.46	−0.99-0.07	0.09	76	0.04
	No	8	426	1710	−0.20	−0.55-0.15	0.27	86	0.00001
24 h	Yes	3	117	380	0.63	0.42–0.84	< 0.00001	0	1
	No	2	94	986	0.33	0.11–0.54	0.003	82	0.02

*Abbreviations No* number, *MI* myocardial infarction, *SMD* standardized mean difference, *NR* unreported, *CI* confidence interval, *ACS* acute coronary syndromes, *CAD* coronary artery disease, *MACEs* major adverse cardiac events

**Table 4:** Source of heterogeneity by meta-regression analysis

Factors	Coefficient	Standard error	*P*
Follow-up duration			
On admission	0.4594606	0.4769995	0.364
24 h	0.7399235	0.1979834	0.033
Type of CAD			
On admission	−0.3537444	0.4880535	0.489
24 h	0.4633034	0.1574912	0.06
PCI			
On admission	−0.2209772	0.6057707	0.725
24 h	0.3064662	0.2227691	0.263
Regular anticoagulant drugs			
On admission	0.3608304	0.5222992	0.509
24 h	0.6721909	0.1000211	0.007
Antiplatelet			
On admission	−0.3252659	0.4913701	0.527
24 h	0.6721909	0.1000211	0.007

### Sensitivity analyses

Each study was excluded sequentially to evaluate the influence of an individual study on the results. No study fundamentally changed the combined effects at any time points. Furthermore, the study by Warlo [10] was found to be the source of heterogeneity. When the study was eliminated from analysis, heterogeneity become minimal (examined on 24 h: SMD = 0.67, 95% CI = 0.47–0.86, *P* < 0.00001, I^2^ = 0%).

### Publication bias

**Fig. 3:**
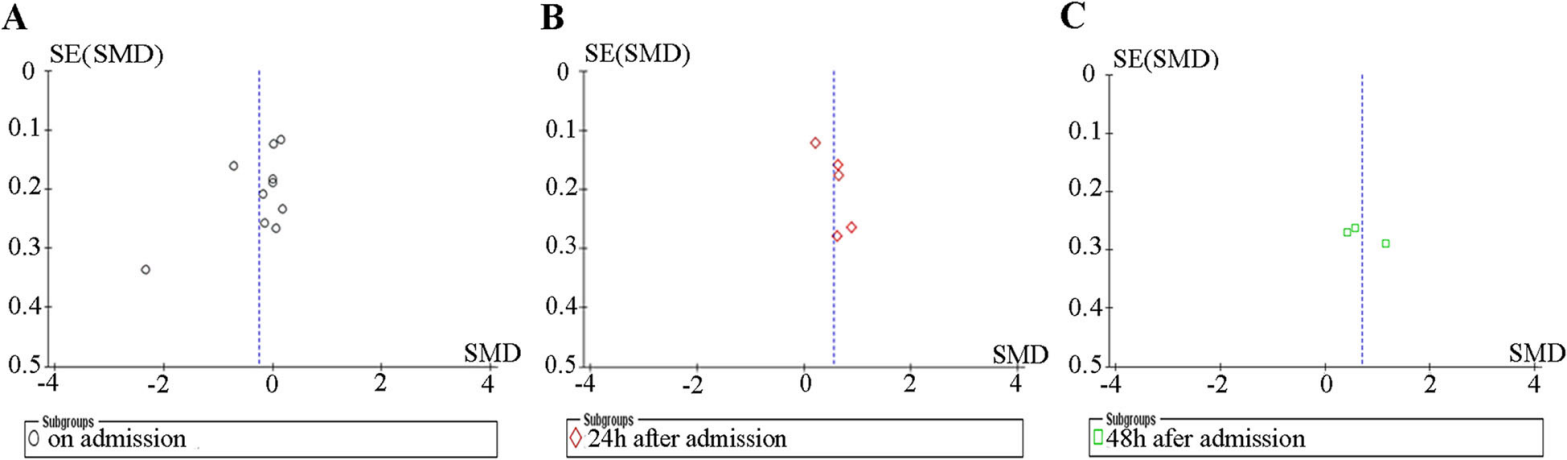
Funnel plot of publication bias. No publication bias was presented in any groups. **a** Funnel plot of studies on admission of vWF levels. **b** Funnel plot of studies at 24 h after admission of vWF levels. **c** Funnel plot of studies at 48 h after admission of vWF levels

Funnel plot was performed to evaluate the publication bias of literatures. As shown in Fig. 3.

## Discussion

This meta-analysis summarizes evidence for association between high-circulation vWF levels and clinically adverse outcomes in patients with CAD. The data on plasma vWF at three time points was included. Results indicated that the plasma vWF was significantly increased in the adverse event group on 24 h and 48 h after primary CAD. However, the level of vWF on admission showed no significant difference between the two groups. Subgroup analyses revealed that the association of increased vWF level with short-term MACEs is stronger. In addition, increased vWF level displays a positive association on MACEs in ACS and MI other than stable CAD. Together, our results suggested that plasma level of vWF is an indicator for the risk of MACEs among patients with CAD.

vWF is mainly synthesized in endothelial cells [25, 26]. Upon endothelial cell injury, vWF is released into the blood circulation. In blood plasma, vWF binds to platelet receptors GPIb-IX-V, GPIIb/IIIa and GPIb to promote thrombosis [27, 28]. The combination of vWF and collagen causes a conformational change in the site of vWF binding to factor VIII, which promotes fibrin agglutination [29, 30]. VWF also mediates platelet adhesion on activated endothelial cells, enhancing thrombus formation even in the absence of endothelial denudation [31]. Several studies have reported that high plasma vWF levels are associated with endothelial dysfunction and inflammation [32, 33], which contribute to the cardiovascular risks. In addition, vWF involves in the pathogenesis of atherosclerosis [34].

MACEs, such as nonfatal myocardial infarction, nonfatal stroke, or target vessel revascularization, are more likely to occur in patients with severe CAD [3]. The increased risk of vWF for MACEs in CAD may be caused by prothrombotic or hypercoagulable conditions, which promote the formation of occlusive thrombus [35]. Both acute myocardial infarction (AMI) and stroke are precipitated by an occlusive thrombus on a preexisting atherosclerotic plaque [36]. VWF promotes thrombus formation by mediating platelet adhesion and aggregation [36]. The inflammatory response involved in the progression of atherosclerotic plaques may also promote an increased secretion of vWF [37]. Therefore, vWF may be considered as a potential clinical biomarker. Previous studies reported that PCI leads to a significant increase of vWF levels compared with the pre-procedural levels [38]. PCI itself causes endothelial cell damage due to mechanical injury by catheter manipulations [39]. In addition, hemodynamic effects of transient myocardial injury during PCI contributes to the increased vWF levels [40].

The prognostic role of vWF in patients with CAD is even more convinced than other acute phase-reactive proteins such as his-CRP and fibrinogen [41]. Studies have shown that elevated early vWF levels in patients with CAD are an independent predictor of adverse events over the next 2 weeks to 1 month, whereas other acute phase response proteins are not [42]. Compared with reactive proteins, vWF is released locally during vascular injury without new synthesis of proteins. Recent case-control studies also demonstrated that the higher plasma vWF or lower ADAMTS13 levels were closely associated with the risk of MI [33, 43–45], ischaemic stroke [46]. However, the present study is the first meta analysis that highlights the long-term prognostic value of plasma vWF levels in patients with CAD.

Our study has several advantages. First, vWF is a promised indicator of the clinical outcome in patients with coronary artery disease. The dramatic increase of plasma vWF implies its potential roles in the diagnosis of CAD. Second, all the studies included in this meta-analysis were medium-to-high quality as assessed by Newcastle-Ottawa Quality Assessment Scale. Third, publication bias assessment confirmed the robustness and reliability of our results. Moreover, circulating vWF level was collected on admission, 24 h and 48 h after primary CAD respectively, which provides a variation of vWF with the progression of disease. Our study has several limitations. First, the number of studies of duration on 24 h or 48 h available for meta analyzes was relatively small. Second, the articles included many types of coronary artery disease including acute coronary syndrome, myocardial infarction, angina, which may contributes to clinical heterogeneity. Third, detailed information regarding symptom duration was not available in several studies.

## Conclusion

Plasma vWF levels of CAD patients examined at 24 h and 48 h after admission might be an independent prognostic factor for MACE. However, many studies had incomplete information, and more studies with more detailed data and sufficient sample size are necessary to confirm our findings.

## Data Availability

All data generated or analyzed in this study are included in this manuscript.

## Abbreviations

95%CI: 95% confidence intervals
ACS: Acute coronary syndromes
AMI: Acute myocardial infarction
CAD: Coronary artery disease
CK-MB: Creatine kinase MB ECG: electro cardio graph
IQR: Inter quartile range
MACEs: Major adverse cardiac events
non-STEMI: Non-ST-elevated myocardial infarction
NOS: Newcastle-Ottawa quality assessment scale
PCI: Percutaneous coronary intervention
SD: Standard deviation
SE: Standard error
SMD: Standard mean difference
STMI: ST-elevated myocardial infarction
vWF: von Willebrand factor
